# Theoretical analysis of headache recurrence in patients administered triptans for migraine based on receptor occupancy

**DOI:** 10.1186/s10194-015-0558-9

**Published:** 2015-08-05

**Authors:** Kentaro Tokuoka, Risa Takayanagi, Mioko Toyabe, Masayuki Watanabe, Yasuhisa Kitagawa, Yasuhiko Yamada

**Affiliations:** Department of Neurology, Tokai University Hachioji Hospital, 1838 Ishikawa-cho, Hachioji, Tokyo 192-0032 Japan; School of pharmacy, Tokyo University of Pharmacy and Life Sciences, 1432-1 Horinouchi, Hachioji, Tokyo 192-0392 Japan; Department of Pharmacy, Tokai University Hachioji Hospital, 1838 Ishikawa-cho, Hachioji, Tokyo 192-0032 Japan

**Keywords:** Triptan, Migraine, Headache recurrence, 5-HT_1B_ receptor, 5-HT_1D_ receptor, Receptor occupancy

## Abstract

**Background:**

In this study, we retrospectively analyzed the relationship between headache recurrence and serotonin 5-HT_1B/1D_ receptor occupancy (Φ_1B_ and Φ_1D_). Triptans marketed in Japan (sumatriptan, zolmitriptan, eletriptan, rizatriptan, naratriptan) were investigated.

**Methods:**

Receptor occupancies were calculated from both the pharmacokinetic and pharmacodynamic data of triptans. We examined the relationships between recurrence rate and elimination half-lives, and Ф_1B_ and Ф_1D_, as calculated from the time-course of plasma drug concentration obtained from other studies. The time until Ф_1B_ and Ф_1D_ became 50 % or less, 40 % or less, and 30 % or less was calculated as duration time to examine the relationship with recurrence rate.

**Results:**

For Ф_1B_, eletriptan remained at a low level. For Ф_1D_, it was indicated that all triptans obtained an occupancy of 80 % or higher at maximum. For all items, though recurrence tended to be lower along with longer half-life, no significant statistical correlation was found. For both Ф_1B_ and Ф_1D_, the recurrence rate tended to be lower as the duration became longer. In addition, a significant correlation was observed for Ф_1D_ (p < 0.05). For clarifying the Ф value and time period most closely correlated with recurrence rate, recurrence and Ф_1B_ and Ф_1D_ at 6, 12, and 18 h after administration were calculated. The most significant correlation was observed between recurrence rate and Ф_1D_ at 12 h after administration (p < 0.01).

**Conclusions:**

As an index for evaluating headache recurrence following triptan administration, recurrence rate and Ф_1D_ value at 12 h after administration were found to be most closely correlated and useful for analysis. Our results indicate that headache recurrence inhibition can be evaluated using these values.

## Background

Triptans are serotonin 5-HT_1B/1D_ agonists frequently used as migraine-abortive drugs. Presently, 5 different triptans are marketed in Japan, though lack of information is a problem for selection of the proper drug and that choice is largely dependent on only the experience of the attending physician. Even in the Japanese ‘Practical guidelines for chronic headaches’, it is noted that the triptans vary in regard to their pharmacological properties and therapeutic effects different with individual patients. For choice of the rational triptan, the only description states that the proper choice has not yet been elucidated, as there have been few closed studies with a satisfactory number of subjects [1]. Furthermore, it has been pointed out that the headache sometimes returned even after administration. Thus, it is considered indispensable to establish a theoretical dosage regimen for inhibiting recurrence. Differences in triptan’s therapeutic effects among individuals and drug characteristics, as well as lack of evidence for selecting a suitable drug for each patient are problems to be resolved. And it is important to establish an index for quantitative evaluation of the pharmacological and clinical effects of triptans.

Concerning headache recurrence following triptan administration, relationships between recurrence and elimination half-lives of plasma drug concentrations have been reported in other countries [2], whereas nearly nothing has been presented on this subject in Japan. Meanwhile, our department performed analysis of the clinical effects of triptans (headache relief rate) by focusing on serotonin 5-HT_1B_ and 5-HT_1D_ receptor occupancies (Ф), and we found that the parameter (*A*_*Ф*_ • AUC_Ф_) of the area under the time curve of Ф (AUC_Ф_) until the efficacy evaluation time point after correction by the velocity factor *A*_*Ф*_ (Ф_max_/T_max_) could serve as an index to correct the differences between drug and formulations, and uniformly evaluate them [3, 4].

In the present study, we performed a theoretical evaluation of headache recurrence following triptan administration in Japanese based on serotonin 5-HT_1B_ and 5-HT_1D_ receptor occupancies.

## Methods

### Collection of pharmacokinetic and pharmacodynamic parameters

Triptans marketed in Japan (sumatriptan, zolmitriptan, eletriptan, rizatriptan, naratriptan) were investigated. Data regarding pharmacokinetic and pharmacodynamics parameters, and headache recurrence rate (referred to as recurrence rate hereinafter) were obtained from published studies. In Japan, data obtained from various clinical trials performed domestically are included in the clinical data package for new drug registration, while drug properties and clinical trial results were extracted from those downloadable from the website of the regulatory authority the Pharmaceuticals and Medical Devices Agency (PMDA) (http://www.pmda.go.jp). When data were not available at that website, they were collected from published studies. For pharmacokinetic parameters, data for plasma drug concentration following administration of each drug, plasma elimination half-life, plasma unbound protein fraction, and the presence of active metabolite were collected from clinical trial results. For pharmacodynamics parameters, the dissociation constant Ki values for the serotonin 5-HT_1B_ and 5-HT_1D_ receptors were obtained.

### Calculation of serotonin 5-HT_1B_ and 5-HT_1D_ receptor occupancies

On the basis of the receptor occupancy theory, the time-course changes of serotonin 5-HT_1B_ and 5-HT_1D_ receptor occupancies Ф (Ф_1B_ and Ф_1D_) of the triptans were calculated.

Triptan can be classified into two types. One is only the unchanged drug has pharmacological effect, and the other is both of unchanged drug and active metabolite has pharmacological effect.

1. When only the unchanged drug was involved in drug efficacy:

The plasma concentration of the unbound drug (C_f_) after a single administration of the drug in Japanese subjects, and the dissociation constant Ki (Ki_1B_ and Ki_1D_) of serotonin 5-HT_1B_ and 5-HT_1D_ were substituted in equation (1) to calculate the time course of Ф_1B_ and Ф_1D_.

1 $$ \Phi\ \left(\%\right) = \frac{{\mathrm{C}}_{\mathrm{f}}}{{\mathrm{C}}_{\mathrm{f}}+\mathrm{K}\mathrm{i}} \times 100 $$

2) When only one kind of active metabolite was present:

The changes in Ф_1B_ and Ф_1D_ were calculated by substituting the plasma unbound drug concentrations (C_f_^1^ and C_f_^2^) of the unchanged drug and active metabolite after a time of single-dose administration, and the dissociation constants of serotonin 5-HT_1B_ and 5-HT_1D_ (Ki_1B_^1^, Ki_1D_^1^ and Ki_1B_^2^, Ki_1D_^2^) in equation (2).

2 $$ \Phi\ \left(\%\right) = \left\{\frac{{\mathrm{C}}_{\mathrm{f}}^1}{{\mathrm{C}}_{\mathrm{f}}^1+{\mathrm{Ki}}^1\left(1+\frac{{\mathrm{C}}_{\mathrm{f}}^2}{{\mathrm{Ki}}^2}\right)}+\frac{{\mathrm{C}}_{\mathrm{f}}^2}{{\mathrm{C}}_{\mathrm{f}}^2+{\mathrm{Ki}}^2\left(1+\frac{{\mathrm{C}}_{\mathrm{f}}^1}{{\mathrm{Ki}}^1}\right)}\right\} \times 100 $$

### Relationship between headache recurrence and serotonin 5-HT_1B_ and 5-HT_1D_ receptor occupancies

The relationships between time-course changes of Ф_1B_ and Ф_1D_ were obtained, and headache recurrence rate (recurrence rate) were examined. For the definition of recurrence, migraine pain was rated as follows: grade 0, no pain; grade 1, mild pain; grade 2, moderate pain; grade 3, severe pain. The patients whose headache improved from grade 3 or 2 to grade 1 or 0 at 1-4 h after initial administration but returned to grade 3 or grade 2 within 24 h after administration were regarded as having recurrence. Data for patients who met the requirements were collected from clinical trial data published in the application summary and published reports. The headache recurrence rate (recurrence rate) was obtained by dividing the number of subjects with recurrence by the number of those with relief. Then the following items (1-3) were examined.

1. Relationships between headache recurrence rate (recurrence rate) and elimination half-lives of plasma drug concentrations, Ф_1B_, and Ф_1D_.
2. Relationships between headache recurrence rate (recurrence rate) and duration time of Ф_1B_ and Ф_1D_.
3. Relationships between headache recurrence rate (recurrence rate) and Ф_1B_ and Ф_1D_ at 6, 12, and 18 h after administration.

## Results

### Extraction of pharmacokinetic and pharmacodynamics parameters for drugs and recurrence rate

Table 1 shows the generic name, formulation, elimination half-life (t_1/2_), molecular weight (MW), plasma unbound fraction (f_u_), and Ki values for the serotonin 5-HT_1B_ and 5-HT_1D_ receptors. For zolmitriptan, data for both the unchanged drug and active metabolite were collected, as an active metabolite that seemingly affected efficacy was considered to be present.

**Table 1:** Pharmacokinetic and pharmacodynamic parameters of triptans [12, 15, 19–24]

Drug (formulation)		t_1/2_ (hr)	MW	f_u_	Ki (nM)	
					5-HT_1B_	5-HT_1D_
Sumatriptan (subcutaneous injection) [19]		1.46	295.40	0.66	12.59	12.59
Zolmitriptan (oral tablet)	Unchanged drug [12, 20]	2.40	287.30	0.75	^a^6.31	^a^2.51
	Active metabolite [12, 20, 21]	2.35	273.36	0.75	^a^1.58	^a^0.50
Eletriptan (oral tablet) [22]		3.20	382.52	0.13	10.0	1.15
Rizatriptan (oral tablet) [23, 24]		1.60	269.35	0.86	7.24	2.34
Naratriptan (oral tablet) [15]		5.05	335.47	0.71	2.24	2.30

^a^Calculated from radioligand test

Table 2 shows the dose and formulation for single-dose administration, and recurrence rate for each of the 5 triptans. The recurrence rate varied depending on the drug and formulation.

**Table 2:** Japanese headache recurrence rate data [12, 24–27]

Drug	Dose (mg/time)	Formulation	Recurrence rate (%)
Sumatriptan [25]	3	Subcutaneous injection	54.8 [n = 31]
Zolmitriptan [12]	2.5	Oral tablet	13.0 [n = 30]
Eletriptan [26]	20	Oral tablet	10.0 [n = 51]
	40	Oral tablet	17.0 [n = 52]
	80	Oral tablet	14.0 [n = 59]
Rizatriptan [24]	10	Oral tablet	31.7 [n = 41]
	10	RPD oral tablet	46.9 [n = 32]
Naratritpan [27]	1	Oral tablet	24.3 [n = 74]
	2.5	Oral tablet	11.9 [n = 84]

*RPD* rapid dissolution

### Time course of Ф after administration of each triptan

**Fig. 1:**
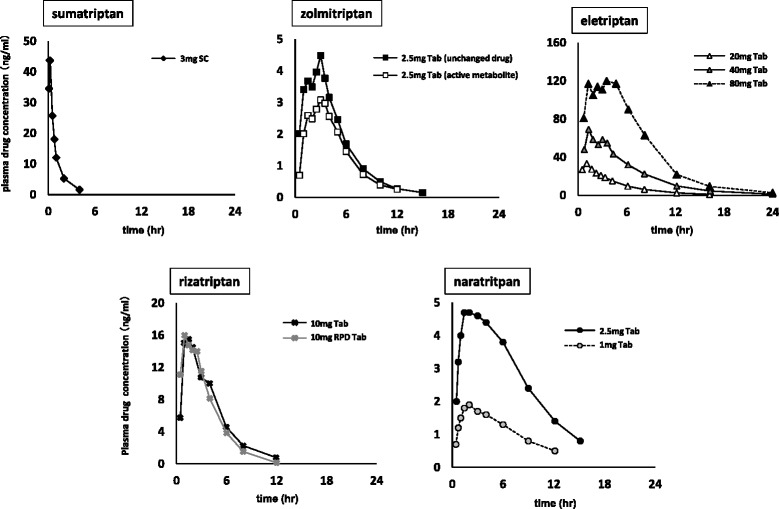
Changes in plasma drug concentrations after a single-dose administration in Japanese subjects. (SC: subcutaneous injection, Tab: oral tablet, RPD Tab: rapid dissolution oral tablet, solid line: usual-dose in Japan)

**Fig. 2:**
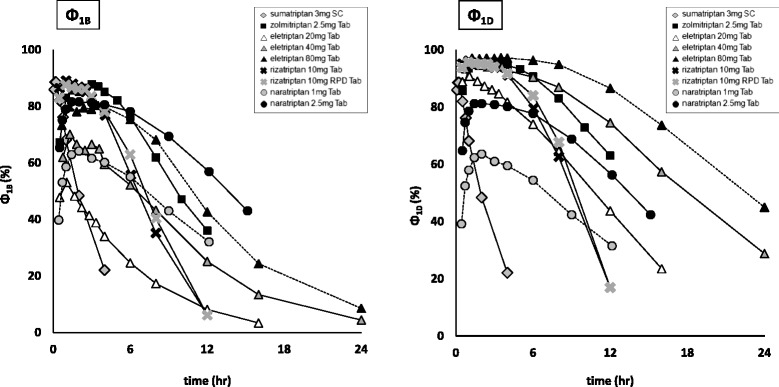
Changes in Ф_1B_ and Ф_1D_ at the time of usual-dose administration. (SC: subcutaneous injection, Tab: oral tablet, solid line: usual-dose in Japan)

Changes in plasma drug concentrations after a single-dose administration for 5 triptans in Japanese subjects are shown in Fig. 1. These data included not only usual but also clinical dose data by which evaluation of recurrence rate was reported. Changes in serotonin 5-HT_1B_ and 5-HT_1D_ receptor occupancies (Ф_1B_ and Ф_1D_) at the time of usual-dose administration calculated from the pharmacokinetic and pharmacodynamic parameters are shown in Fig. 2.

For Ф_1B_, eletriptan remained at a low level. For Ф_1D_, it was indicated that all triptans obtained an occupancy of 80 % or higher at maximum. Moreover, the injectable form of sumatriptan showed a quicker occupancy decline than the other triptans for Ф_1B_ and Ф_1D_.

### Relationship with recurrence rate

#### Relationship between recurrence rate and elimination half-lives of plasma drug concentrations Ф_1B_ and Ф_1D_

**Fig. 3:**
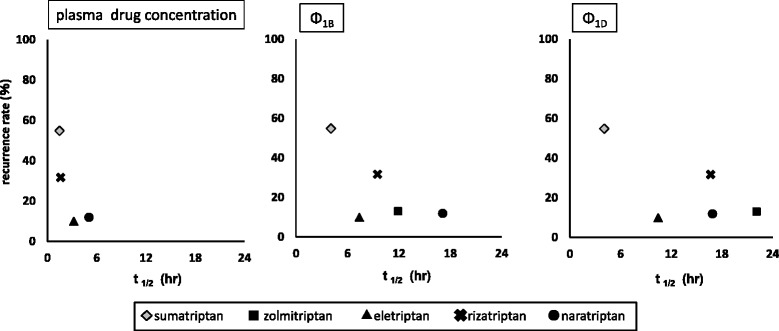
Relationships between headache recurrence rate and elimination half-lives of plasma drug concentration, Φ_1B_ and Φ_1D_

The relationships between recurrence rate and elimination half-lives, and Ф_1B_ and Ф_1D,_ as calculated from the time-course of plasma drug concentration obtained from other studies, are shown in Fig. 3. In our investigation of elimination half-life, zolmitriptan with active metabolites was excluded, as it could not be examined only on the basis of half-life in an unchanged drug.

For all items, though recurrence tended to be lower along with longer half-life, no significant statistical correlation was found.

#### Relationship between recurrence rate and duration time of Ф

The time until Ф_1B_ and Ф_1D_ became 50 % or less, 40 % or less, and 30 % or less was calculated as duration time to examine the relationship with recurrence rate. Table 3 shows the calculated duration time of Ф_1B_ and Ф_1D_.

**Table 3:** Calculated duration time of Ф

[Φ_1B_]				
Drug	Dose (mg/time) (formulation)	Time until Ф_1B_ became 50 % or less (hr)	Time until Ф_1B_ became 40 % or less (hr)	Time until Ф_1B_ became 30 % or less (hr)
Sumatriptan	3 (subcutaneous injection)	1.3	2.4	2.5
Zolmitriptan	2.5 (oral tablet)	9.6	11.3	13.1
Eletriptan	20 (oral tablet)	1.4	3.1	6.1
	40 (oral tablet)	6.7	8.9	11.1
	80 (oral tablet)	11	12.8	14.9
Rizatriptan	10 (oral tablet)	8	9.6	11.3
	10 (RPD oral tablet)	7.2	8.1	9.2
Naratritpan	1 (oral tablet)	7.2	9.8	12.7
	2.5 (oral tablet)	13.7	15.8	18
[Φ_1D_]				
Drug	Dose (mg/time) (formulation)	Time until Ф_1D_ became 50 % or less (hr)	Time until Ф_1D_ became 40 % or less (hr)	Time until Ф_1D_ became 30 % or less (hr)
Sumatriptan	3 (subcutaneous injection)	1.3	2.4	2.5
Zolmitriptan	2.5 (oral tablet)	14.6	16.6	18.6
Eletriptan	20 (oral tablet)	10.9	12.8	14.9
	40 (oral tablet)	18.3	21.2	24
	80 (oral tablet)	22.9	26.7	31.1
Rizatriptan	10 (oral tablet)	12.2	13.8	15.5
	10 (RPD oral tablet)	9.3	10.1	10.8
Naratritpan	1 (oral tablet)	7.2	9.7	12.7
	2.5 (oral tablet)	12.4	15.6	17.9

*RPD* rapid dissolution

**Fig. 4:**
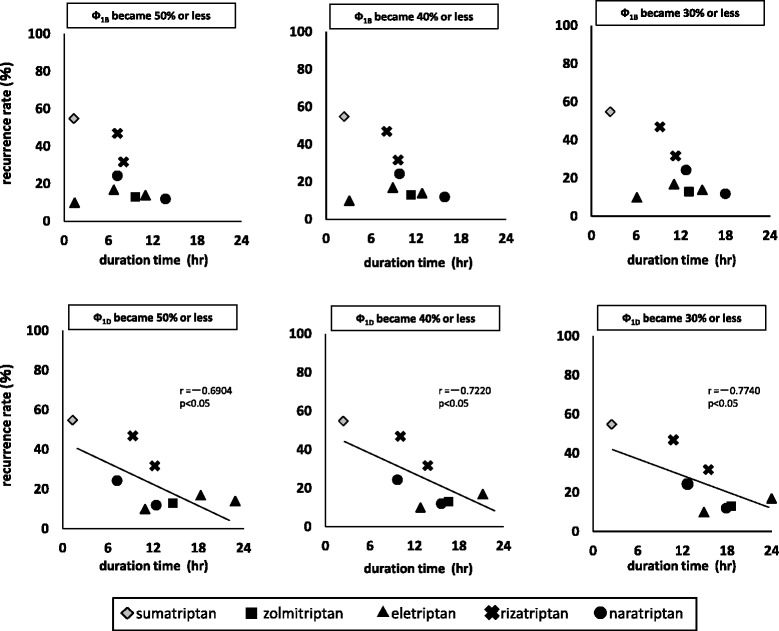
Relationships between headache recurrence and duration time of Φ_1B_ and Φ_1D_

Figure 4 shows the relationship with the duration time of Ф_1B_ and Ф_1D_.

For both Ф_1B_ and Ф_1D_, the recurrence rate tended to be lower as the duration became longer. In addition, a significant correlation was observed for Ф_1D_.

#### Relationship between recurrence rate and Ф_1B_ and Ф_1D_ at 6, 12, and 18 h after administration

For clarifying the Ф value and time period most closely correlated with recurrence rate, recurrence and Ф_1B_ and Ф_1D_ at 6, 12, and 18 h after administration were calculated. Table 4 shows calculated Ф_1B_ and Ф_1D_ values at 6, 12, and 18 h after administration.

**Table 4:** Calculated Ф values at 6, 12, and 18 h after administration

[Φ_1B_]				
Drug	Dose (mg/time) (formulation)	6 h after administration (%)	12 h after administration (%)	18 h after administration (%)
Sumatriptan	3 (subcutaneous injection)	0	0	0
Zolmitriptan	2.5 (oral tablet)	76.3	36.1	3.3
Eletriptan	20 (oral tablet)	25.3	8.7	1.5
	40 (oral tablet)	52.9	25.7	11.4
	80 (oral tablet)	75.7	43.6	20.7
Rizatriptan	10 (oral tablet)	66.9	25.8	0
	10 (RPD oral tablet)	62.9	5.7	0
Naratritpan	1 (oral tablet)	54.3	32.1	10
	2.5 (oral tablet)	78.2	57.7	57.7
[Φ_1D_]				
Drug	Dose (mg/time) (formulation)	6 h after administration (%)	12 h after administration (%)	18 h after administration (%)
Sumatriptan	3 (subcutaneous injection)	0	0	0
Zolmitriptan	2.5 (oral tablet)	90.7	63.1	33
Eletriptan	20 (oral tablet)	74.3	44.3	14.7
	40 (oral tablet)	90.7	75	50.7
	80 (oral tablet)	96.4	87.1	67.1
Rizatriptan	10 (oral tablet)	86.2	51.8	15
	10 (RPD oral tablet)	83.6	16.4	0
Naratritpan	1 (oral tablet)	54.3	30.3	11.4
	2.5 (oral tablet)	77.8	57	29.3

*RPD* rapid dissolution

**Fig. 5:**
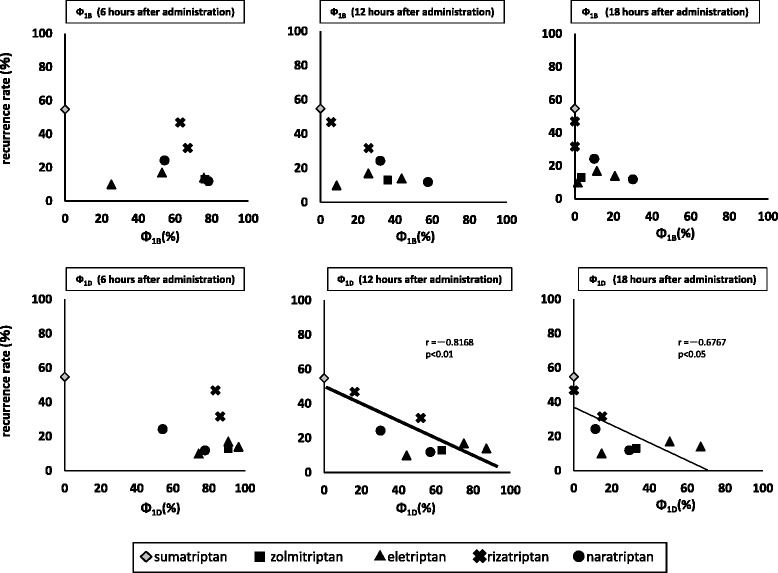
Relationships between headache recurrence and Φ_1B_ and Φ_1D_ at each time after administration

The relationships between recurrence rate and Ф_1B_ and Ф_1D_ at each time point are shown in Fig. 5.

For Ф_1B_, the aspect differed depending on each time point, while for Ф_1D_, the recurrence rate tended to be lower when Ф_1D_ was larger. Significant correlations with recurrence rate were observed at 12, and 18 h after administration. Notably, the most significant correlation was observed between recurrence rate and Ф_1D_ at 12 h after administration.

## Discussion

During migraine treatment, a headache sometimes returns after remission achieved by administration of triptans, which are serotonin 5-HT_1B/1D_ receptor agonists. Accordingly it is indispensable to establish information related to proper use to prevent recurrence. In the present study, we performed theoretical evaluations for establishing proper use by calculating serotonin 5-HT_IB_ and 5-HT_1D_ receptor occupancies, and examined their relationship with headache recurrence.

Drug action is determined by drug concentration in the region of the site of action and binding to the target receptor. When drug action is elicited by a specific target receptor, it is important to kinetically clarify the relationship between them. In our previous study, we have reported that receptor occupancy is useful for theoretical evaluation of the standard therapeutic doses of various groups of drugs, and a more appropriate index for estimating drug action than dose or blood concentration [5–10].

For collecting pharmacokinetic parameters and recurrence data, we selected only clinical trials that used Japanese subjects. Regarding pharmacodynamic parameters, we collected Ki values that showed an affinity to the serotonin 5-HT_1B_ and 5-HT_1D_ receptors based on pharmaceutical interview forms and literature from new drug application data summaries. Only for zolmitriptan, the Ki value could not be obtained from literature. However, it was reported that the Ki value was equivalent to the IC_50_ value of the radioactive ligand displacement trial [11], thus we used it as substitute for the Ki value in our analysis. Furthermore, since one kind of active metabolite considered to affect efficacy was present for zolmitriptan, we collected data regarding the unchanged drug and active metabolite for the present analysis.

In this study, therefore using the pharmacokinetic data of a Japanese subject, it was necessary to examine the data for clinical trials in Japan. However, the information that has been published in clinical trials of Japanese was less. In the future, in order to analyze in more detail, it is required to include more data for clinical trials.

The definition of recurrence was found to vary among the clinical trials examined. Notably, the method for determining the time period until headache relief following the initial administration differed. In the present analysis, for comparing recurrence rates among the 5 triptans according to the same definition, we used the following: the ratio of patients who had headache relief 1-4 h after the initial administration but had headache return within 24 h after administration.

In clinical trials of zolmitriptan, there was no difference found in regard to recurrence rate between patients who had headache relief 2 h after administration and those who had relief 4 h after administration [12]. Accordingly, we employed that in our analysis based on the assumption that the method for determining the time period until headache relief may not cause a dispersion in recurrence rate. In the future, it is expected that analysis precision might be further improved if comparisons can be made by using data collected according to a unified definition of recurrence. Using our definition to analyze clinical trial data of Japanese patients we obtained recurrence rates for sumatriptan (3 mg subcutaneous injectable) (headache relief at 1 h after administration), zolmitriptan (2.5-mg tablets), eletriptan (20-, 40-, 80-mg tablets), rizatriptan (10-mg tablets), RPD (10-mg tablets) (headache relief at 2 h after administration), and naratriptan 1- and 2.5-mg tablets (headache relief at 4 h after administration). The rate was relatively low for eletriptan and naratriptan, whereas it was high for rizatriptan. Also, the injectable form had a higher rate of recurrence than tablets. Thus, our findings clarified that recurrence rate varied among the kind and administered form of the drugs.

In the present analysis, the drug concentration near the subject receptor was speculated to be at the same level as that of the plasma unbound drug concentration. Triptans act on the serotonin 5-HT_1B_ and 5-HT_1D_ receptors, and they are thought to work in three main ways: 1) peripheral inhibition of release of vasodilator neuropeptides; 2) modulation of second-order neurons centrally in the trigeminocervical pathway; and 3) vasoconstriction [13]. There are no studies that performed direct measurements of drug concentration near those receptors, thus we speculate that the drug transits to plasma after administration and the unbound form from plasma protein penetrates through the vascular wall to reach the action site [14]. Smatriptan and naratriptan especially are scarcely passed through the blood–brain barrier (BBB) and were weakly distributed in the central nervous system [15, 16]. However, they elicited the same therapeutic effect for migraine as other triptans. Furthermore, it is reported that triptans also acted on 5-HT_1B/1D_ receptors in the trigeminal and dorsal root ganglion cells where BBB was lacked rather than in the sites with BBB in the trigeminovascular system [17]. Accordingly, we did not take concentration in other tissues such as the brain into consideration. As certain findings could be obtained on the basis of these speculations, we considered that they had some validity. More detailed analysis would be possible if the actual concentration near the receptors could be measured at the time of administration in human subjects.

On the basis of data collected regarding changes in plasma drug concentration in Japanese patients, as well as pharmacokinetic and pharmacodynamics parameters, we calculated the time-course changes of receptor occupancies (Ф_1B_ and Ф_1D_). As the dose varied depending on the kind of triptan, it was considered difficult to make a quantitative evaluation on the basis of plasma drug concentration. However, that of recurrence rate using Ф was considered possible and applicable as a unified index irrespective of drug kind. For Ф_1B_, eletriptan maintained a low level in whole, whereas for Ф_1D_, it was observed that all of the tested triptans attained an occupancy of 80 % or higher at maximum. As compared to the other triptans, sumatriptan injectable had a short half-life of plasma drug concentration, and showed a quicker decline of occupancy for both Ф_1B_ and Ф_1D_.

As for headache recurrence following triptan administration, a study conducted with western subjects noted that a certain relationship could be observed between elimination half-life and recurrence rate [2]. Thus, we first investigated the relationship between recurrence rate and elimination half-life of plasma drug concentration. For both, longer half-life was related to lower recurrence rate, though the differences were not statistically significant. In addition, the drug which has an active metabolite like zolmitriptan, could not be compared with other drugs based on elimination half-life of plasma drug concentration.

In Japan, there was a report which considered the relationship between efficacy time of triptans and recurrence rate [18]. Thus, we examined the relationship between recurrence rate and duration time of Ф after administration. As the Ф_1B_ value of eletriptan was maintained at the relatively low level of 53.1 % at a maximum, our analysis was performed by setting the index of duration time as the time until Ф_1B_ and Ф_1D_ became 50 % or less, 40 % or less, and 30 % or less. For Ф_1B_, no statistically significant correlation could be obtained. For Ф_1D_, recurrence rate tended to be lower as duration time was longer, which showed a statistically significant correlation. Thus, it considered likely that the time required to maintain Ф_1D_ higher than a certain level might have an effect on recurrence rate.

Furthermore, for elucidating the Ф value and time period most closely correlated with recurrence rate, we examined the relationship between recurrence rate and Ф at predetermined time points after administration in subjects who had headache recurrence within 24 h after administration. However, for plasma drug concentration, time-course data up to 24 h after administration could not be calculated, as they were not available. Accordingly, we focused on Ф until 6-18 h after administration, and analyzed the relationship between recurrence rate and Ф every 6 h. No correlation was found between Ф_1B_ and recurrence rate, whereas a significant correlation was observed between Ф_1D_ and recurrence rate, with the most notable at 12 h after administration. On the basis of these findings, it is suggested that higher Ф_1D_ at 12 h after administration is related to lower recurrence rate.

## Conclusions

Based on our findings in this study of receptor occupancy, it is suggested that Ф_1D_ exerts a greater effect on headache recurrence rate after triptan administration in Japanese subjects as compared to Ф_1B_ and that recurrence may be inhibited to a greater degree when Ф_1D_ is maintained at a higher level. Notably, as an index for evaluating headache recurrence following triptan administration, recurrence rate and Ф_1D_ value at 12 h after administration were found to be most closely correlated and useful for analysis. Our results indicate that headache recurrence inhibition can be evaluated using these values.

## Abbreviations

5-HT: 5-hydroxytriptamine
AUC: Area under the curve
Φ: Receptor occupancy
